# Revolutionizing Medicinal Chemistry: The Application of Artificial Intelligence (AI) in Early Drug Discovery

**DOI:** 10.3390/ph16091259

**Published:** 2023-09-06

**Authors:** Ri Han, Hongryul Yoon, Gahee Kim, Hyundo Lee, Yoonji Lee

**Affiliations:** College of Pharmacy, Chung-Ang University, Seoul 06974, Republic of Korea

**Keywords:** artificial intelligence, drug discovery, medicinal chemistry, structure-based drug design

## Abstract

Artificial intelligence (AI) has permeated various sectors, including the pharmaceutical industry and research, where it has been utilized to efficiently identify new chemical entities with desirable properties. The application of AI algorithms to drug discovery presents both remarkable opportunities and challenges. This review article focuses on the transformative role of AI in medicinal chemistry. We delve into the applications of machine learning and deep learning techniques in drug screening and design, discussing their potential to expedite the early drug discovery process. In particular, we provide a comprehensive overview of the use of AI algorithms in predicting protein structures, drug–target interactions, and molecular properties such as drug toxicity. While AI has accelerated the drug discovery process, data quality issues and technological constraints remain challenges. Nonetheless, new relationships and methods have been unveiled, demonstrating AI’s expanding potential in predicting and understanding drug interactions and properties. For its full potential to be realized, interdisciplinary collaboration is essential. This review underscores AI’s growing influence on the future trajectory of medicinal chemistry and stresses the importance of ongoing synergies between computational and domain experts.

## 1. Introduction

Artificial intelligence (AI), a field within computer science, focuses on developing methods that empower computers to perform tasks typically associated with human intelligence, such as thinking and learning. AI is having a revolutionary impact on various facets of our lives and spanning across numerous industry sectors, with the pharmaceutical sector experiencing no exception to this transformation [1]. Also, in the medical field, deep learning techniques classify lung cancer with high accuracy, and AI addresses challenges in processing continuous streams of big data from medical IoT devices [2,3]. The emergence of AI has ushered in a new era in drug discovery research, delivering a paradigm shift from traditional trial-and-error-based or hypothesis-driven methods toward more rational and data-driven approaches [1]. The value of AI is immense as it serves as a technology that can significantly reduce the extensive time and financial investments required for the discovery of a new drug.

When used properly, AI technologies can help analyze vast amounts of data, such as genomic, proteomic, and chemical information, to identify potential drug molecules and predict drug efficacy or toxicity [4]. By analyzing complex datasets and identifying hidden patterns, machine learning (ML) or deep learning (DL) algorithms can find novel targets associated with multi-omics data and help search for novel chemical entities with biological activities. They have not only expedited the identification of potential drug candidates but have also proven invaluable in the process of drug repurposing [5]. AI can predict potential new uses for existing drugs, a breakthrough that has the potential to accelerate the drug development process and reduce associated costs [5]. This capability is particularly significant in addressing urgent medical needs, as repurposing existing drugs can bypass lengthy and costly phases of preclinical testing and safety evaluation. Moreover, AI has emerged as a key tool for personalized medicine by aiding the development of drugs that are tailored to individual patients’ genetic profiles. In the future, the demand for AI in drug discovery is expected to grow as the technology becomes more advanced and many more data become available [1]. 

In the realm of medicinal chemistry, AI has shown promising results in the discovery of new chemical scaffolds with therapeutic potential. It has the capacity to scrutinize vast chemical spaces and extract meaningful patterns, thereby significantly reducing the time required for identifying potential drug candidates [4,6]. ML/DL algorithms can be trained to predict the biological activities, pharmacokinetic properties, and also toxicity profiles of molecules [7]. In addition, the current DL methods can generate novel molecular structures that match desired therapeutic profiles [8,9]. Building on the past decade’s remarkable advances, AI is now being harnessed to automate the process of drug design. Molecular docking, the method that predicts the interaction between a small molecule and a protein, has traditionally been a computationally intensive task. Today, AI is being utilized to predict the likelihood of molecular binding, its strength, and the most energetically favorable position, thereby automating this critical process. In addition, it can be utilized to optimize the chemical structures of drug candidates for enhanced efficacy and reduced toxicity.

While the promise of AI in medicinal chemistry is profound, the integration of AI into drug discovery pipelines presents ongoing challenges [4,10]. Issues related to the quality and availability of data, interpretability of AI models, and regulatory considerations persist [4]. However, as we navigate the ongoing digital transformation, it becomes increasingly evident that AI-based approaches hold immense potential to revolutionize drug discovery and reshape the field of medicinal chemistry. By leveraging AI’s capabilities and addressing the associated challenges, we can harness the power of this technology to accelerate the discovery of safe and effective molecules, ultimately revolutionizing the drug discovery process. 

In this article, we provide a comprehensive review of the state-of-the-art technologies that employ AI in medicinal chemistry. We explore the current advancements and future prospects in this rapidly evolving field, shedding light on the transformative role of AI and its potential impact on drug discovery. The main purpose of this article is not only to outline the breakthroughs AI has facilitated but also to critically evaluate where it falls short or poses new challenges. By considering both the promises and pitfalls of AI in this domain, we aim to offer a balanced perspective that will guide future endeavors. Through a holistic understanding of the state of AI in drug discovery, we aspire to foster a foundation for its more robust and insightful application in the efficient and innovative drug discovery. 

## 2. AI/ML Algorithms and Bio Big Data Utilized in Drug Discovery Research

Bio big data encompass a wide range of data types, such as genomic, proteomic, and transcriptomic data, collected from various sources such as high-throughput experiments and clinical studies, providing invaluable insights for drug discovery [6]. AI/ML algorithms, which are computational methods that allow computers to learn from data and recognize patterns, help researchers navigate these vast amounts of bio big data and identify potential drug candidates more effectively and accurately, revolutionizing the drug discovery process [4,11]. In order to optimally exploit AI and ML strategies within the context of drug discovery, it is essential to grasp the fundamental principles that underpin a range of machine learning methodologies. These methodologies, including supervised, unsupervised, and reinforcement learning, are employed to address a diverse array of research challenges within this domain.

### 2.1. Overview of ML Algorithms

AI is a broad field that encompasses various computational techniques, enabling machines to mimic human-like intelligence capabilities, such as learning, reasoning, and problem-solving. ML is a subset of AI specifically focusing on the development of algorithms that learn, adapt, and perform tasks through data processing and analysis [12]. By identifying patterns, making predictions, and refining algorithms based on input data, ML allows machines to improve their prediction performance and decision-making capabilities autonomously over time. ML algorithms can be broadly categorized into three types: supervised learning, unsupervised learning, and reinforcement learning.

#### 2.1.1. Supervised Learning

**Figure 1:**
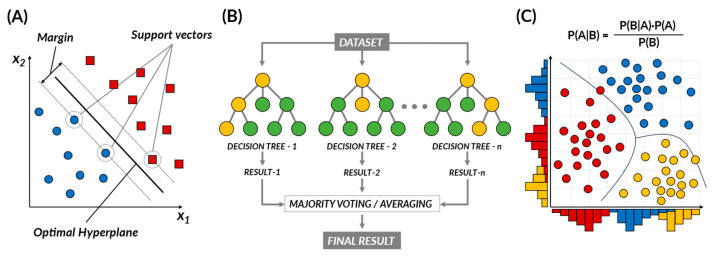
Representative supervised learning algorithms. (**A**) Support vector machine. (**B**) Random forest. (**C**) Naïve Bayes.

Supervised learning is a type of machine learning where algorithms are trained using labeled data, meaning each input data sample is paired with the appropriate or correct output [12]. The algorithm uses these input–output pairs to learn a model that can make accurate predictions for new, unseen data. Supervised learning algorithms, such as support vector machine (SVM), support vector regression (SVR), naïve Bayes, tree-based, and random forest (RF), can identify potential drug candidates by analyzing large datasets and identifying patterns and relationships that humans may not easily detect (Figure 1) [13,14].

(1) *Support Vector Machine (SVM)*

The support vector machine (SVM) is a powerful tool, rooted in the principle of structural risk minimization, and is capable of classifying data, identifying outliers, and performing regression analysis. The core of the SVM methodology is the identification of an optimal decision boundary (i.e., hyperplane) that best separates data points across different classes [15]. This hyperplane is constructed by maximizing the margin, which represents the distance between the decision boundary and the closest training samples, also referred to as support vectors. In drug discovery, SVM is primarily employed to predict the biological activity of compounds or to classify molecular properties. One of the key strengths of SVM in such tasks is its ability to handle high-dimensional data and detect complex patterns, particularly within large and noisy datasets [16,17,18]. This makes SVM one of the top performers in predicting chemical and biological properties. However, it is crucial to note that SVM’s performance can be sensitive to the selection of the kernel function and its parameters [19]. Additionally, when dealing with imbalanced datasets, where one class significantly outnumbers the other, SVM may require additional processing steps to balance the data before application [19].

SVMs have established themselves as a significant tool in drug discovery due to their superior ability to analyze complex cheminformatics data. Their use extends to various tasks: they help in virtual screening processes [20,21], predicting drug–target interactions [22,23], and identifying new drug targets [24,25]. They are instrumental in predicting drug similarity in the quantitative structure–activity relationship (QSAR) domain, where the assumption is that structurally similar compounds will exhibit similar drug activities [23,26]. Furthermore, SVMs are employed to forecast activity cliffs, pairs of structurally similar compounds with a significant activity difference towards a specific target, thereby contributing to our understanding of critical drug–target interactions and aiding in new drug development [16,17,18].

(2) *Naïve Bayes*

The naïve Bayes algorithm is a probabilistic machine learning model rooted in Bayes’ theorem, a principle in probability theory that describes how to update the probabilities of hypotheses when given evidence [12,14]. Specifically, Bayes’ theorem is a mathematical principle that provides a way to update the probabilities of our previous hypotheses based on new evidence [14]. In other words, when new information is given, it helps us decide how to apply it to a hypothesis. The naïve Bayes algorithm takes this principle and applies it with a “naïve” assumption of conditional independence between features [27]. Essentially, it considers each data attribute as independent, thus, simplifying multivariate problems into separate univariate issues. This strong assumption simplifies computations and enables the handling of high-dimensional data with ease. In practice, naïve Bayes has been widely adopted in various fields, such as document analysis, spam detection, and cheminformatics, particularly in drug discovery and drug–target interaction prediction [14]. It is noted for its robustness and versatility. However, despite its simplicity and speed, naïve Bayes has its limitations. It assumes that the attributes in the dataset are entirely independent, which might not accurately reflect the actual dependencies present in the data. Moreover, while naïve Bayes performs reasonably well as a classifier, it is known to be a less reliable probability estimator, so its output probabilities should be interpreted cautiously.

In drug design, the naïve Bayes algorithm has been widely applied, helping predict the biological activities of compounds, assisting in the early selection of promising candidates, and estimating results before laboratory experiments [7,28]. It can predict protein–protein [29] and drug–drug interactions [30], which is vital for understanding cellular pathways and managing polypharmacy, where patients take multiple drugs. This algorithm can also anticipate drug–target interactions, facilitating drug repurposing and side effect prediction [31,32,33,34]. Lastly, it can classify compounds into specific categories quickly, although it operates on the assumption of feature independence, which may not always hold [27,35].

(3) *Random Forest (RF)*

Tree-based ML algorithms use decision trees (DTs) to predict target values based on observed features. DTs are flowchart-like structures where each internal node represents a feature, branches represent decision rules, and leaf nodes indicate outcomes, allowing for classification and regression tasks. However, single decision trees are prone to overfitting and struggle to generalize to new data. To overcome this limitation, ensemble methods, such as random forest (RF), prove to be particularly beneficial [12,13] The RF algorithm creates an ensemble of DTs, each built on a different sample of the data [14]. Each split in these DTs is determined from a different subset of features, leading to decorrelation between the trees. This strategy combats the overfitting problem often encountered with single DTs. By aggregating results from numerous, ideally uncorrelated, DTs, RF leverages the power of ensemble learning, enhancing its predictive power and stability. RF provides benefits in early drug discovery, including enhanced feature selection and predictive ability in QSAR analysis, making it useful for handling large, high-dimensional datasets in virtual screening [36]. However, to manage overfitting risks, careful data partitioning, model complexity control, and cross-validation are necessary. Analyzing feature importance can improve interpretability [12].

Building on these strengths, RF has been integrated into various stages of drug development, such as predicting chemical and drug properties, protein-related predictions, conducting virtual screening and docking studies, drug response prediction, polypharmacology research, and drug side effects prediction. Specifically, RF has proven helpful in QSAR modeling to correlate a drug’s chemical structure with its biological activity, estimating key parameters such as drug solubility and solvent density [36]. In protein-related predictions, RF assists in determining protein pKa values and protein–protein affinity, as well as identifying protein function and type, which is vital in target-based drug design [37]. Additionally, RF models facilitate efficient virtual screening of compound libraries to predict potential binding with target proteins, making them indispensable in integrated virtual screening and docking studies, including peptide docking studies [38].

#### 2.1.2. Unsupervised Learning

**Figure 2:**
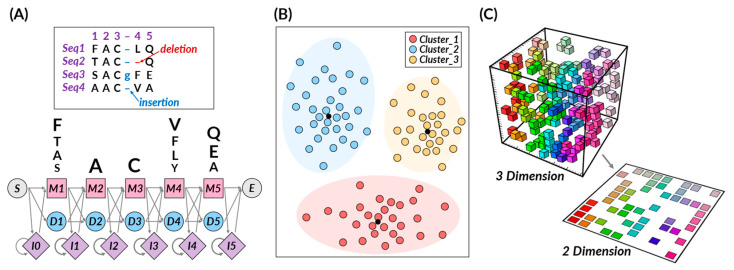
Representative unsupervised learning algorithms. (**A**) Hidden Markov models. (**B**) K-means clustering. (**C**) T-distributed stochastic neighbor embedding.

Unsupervised learning is a method that trains a machine in the absence of any correct answers. It traverses unlabeled data, striving to decipher latent patterns or structures devoid of pre-defined output [5]. In this case, the learning process often involves grouping vast amounts of data based on similar characteristics, a process known as clustering. Even though the correct answer for the input value is not known, unsupervised learning can be used to uncover hidden patterns or features within the data, making it a powerful strategy for clustering and dimensionality reduction. Unsupervised learning algorithms, encompassing hidden Markov models (HMMs), growing self-organizing maps (GSOMs), k-means clustering, principal component analysis (PCA), autoencoders, and t-SNE, exhibit the capacity to cluster similar molecules, unearth novel molecular scaffolds, or reveal previously unknown correlations between biological entities (Figure 2).

(1) *Hidden Markov Models (HMMs)*

Hidden Markov models (HMMs) are probabilistic models developed to work with sequential data. These models rely on a set of unobserved, hidden states and the probability of observable outputs that each state generates [39]. In a specific state, an outcome or observation can be produced according to an associated probability distribution, making HMMs an instrumental tool for various applications. HMMs excel at processing sequential data, modeling temporal dependencies, and managing missing and noisy data, making them robust against overfitting due to their probabilistic nature [40]. However, since HMMs rely on the Markov assumption, asserting that future states depend only on the current state, it might not always be a realistic presumption for many real-world scenarios.

HMMs have proven to be an invaluable tool in drug discovery, primarily due to their prowess in analyzing sequential biological data. They find extensive applications in various pivotal tasks. HMMs are critical in protein homology detection, efficiently identifying and classifying protein families within sequences [41,42]. This ability is vital in discovering new proteins that serve as potential targets for novel drugs. Further, HMMs play a significant role in protein sequence analysis, a critical process in understanding the function of a protein and selecting it as a target in the early stages of drug development [43,44]. Through augmenting sequence analysis, HMMs reveal more accurate and profound insights, thereby enhancing the outcomes. Lastly, HMMs are instrumental in predicting protein structures and 3D modeling. Accurately predicting and modeling complex protein structures is a crucial part of drug discovery, as it assists in predicting the efficacy and binding characteristics of a potential drug [45].

(2) *K-means Clustering*

K-means clustering is a powerful tool, grounded in the partitioning principle, adept at classifying data into distinct ‘k’ clusters. A centroid, the mean of all data points within that cluster, characterizes each cluster. This algorithm aims to assign each data point to the nearest cluster, creating homogeneous groups of similar data points [1]. In drug discovery, k-means clustering is primarily used to define proper molecular descriptors, compute the similarities between compound samples, and group compound features based on computed similarities. A key strength of k-means in drug development is its ability to handle high-dimensional data and discern complex patterns, particularly within large and noisy datasets. This capability makes k-means one of the foremost performers in predicting chemical and biological properties. However, it is essential to note that k-means performance can be sensitive to the initial selection of centroids and the predetermined number of clusters. Furthermore, when dealing with imbalanced datasets, k-means may require additional pre-processing steps to balance the data before application. Despite these challenges, the simplicity, scalability, and flexibility of the k-means algorithm make it a vital tool in drug discovery.

Building upon the basic principles, k-means clustering finds extensive use in drug discovery, primarily due to its ability to handle multidimensional data. It helps define molecular descriptors, numerical entities that represent a compound’s physicochemical properties, thereby aiding in predicting its behavior [46,47]. The technique is also proficient in calculating similarities between compound samples, revealing relationships among compounds, and selecting potential drug candidates [48,49]. In addition, k-means clustering is used for clustering compound properties and selecting protein structures based on similarities [46,48,50]. Such grouping helps analyze a drug’s effect, and by identifying similar protein conformations, it enhances the performance of ensemble docking [51,52].

(3) *T-Distributed Stochastic Neighbor Embedding (t-SNE)*

T-distributed stochastic neighbor embedding (t-SNE) is a technique that simplifies high-dimensional data into a more digestible, low-dimensional form while preserving the relative similarities of data points [1]. In short, t-SNE evaluates the similarity of data points in a high-dimensional space, giving higher probabilities to those more similar [53,54]. It maps these points to a lower-dimensional space, aiming to keep these similarities intact [1,53]. The ultimate goal is to create an easier-to-understand visualization while respecting the original data structure. A key advantage of t-SNE is its unique ability to maintain local and global high-dimensional data structures, unveiling patterns other reduction techniques such as PCA might overlook [54]. While t-SNE is effective for visualizing data, it does have limitations. It requires calculating pairwise similarities for all data points, which can be computationally demanding for large datasets [55]. Also, it often struggles to identify relevant clusters at varying scales and is sensitive to hyperparameters, necessitating careful tuning.

As a result, t-SNE plays a central role in drug design, particularly in compound clustering, drug target exploration, molecular representation, and drug design. t-SNE enables a comprehensive understanding and analysis of complex biological data and compound similarity by visualizing high-dimensional data in low-dimensional space. In particular, t-SNE assists in predicting the behavior of compounds through molecular descriptors, which are unique physicochemical characteristics, ultimately playing a crucial role in selecting potential drug candidates [56]. Moreover, t-SNE is employed to visualize biological data, aiding in the understanding of the relationship between drugs and their targets [57,58]. This can help discover new drug targets or identify new uses for existing drugs. Lastly, in visualizing complex biological data such as protein structures and gene expression profiles in lower dimensions, t-SNE enhances the technical aspects of molecular representation and drug design [59]. Due to its versatility and efficiency, t-SNE is expected to continue to play an essential role in shaping drug development strategies.

#### 2.1.3. Reinforcement Learning

Reinforcement learning, a unique branch of machine learning, fine-tunes decision-making strategies through rewards or penalties for each action. Akin to learning via trial and error, a reward is given when the desired result is achieved, and the machine is trained to maximize this reward. If supervised learning and unsupervised learning proceed in a given static environment with the provided data, reinforcement learning, on the other hand, includes the process of collecting data in a dynamic environment. Reinforcement learning algorithms such as Q-learning and Monte Carlo tree search (MCTS) started to be used to revolutionize processes such as molecular docking, de novo drug design, and drug property optimization. These algorithms navigate molecular configurations, assist in constructing novel drug molecules, and balance objectives such as efficacy and side effects to produce promising drug candidates. It can aid the discovery of effective drug molecules and novel therapeutic strategies in a more creative, innovative way.

For example, Q-learning, a specific application of reinforcement learning, optimizes decisions through an intricate balance of ‘exploration’ and ‘exploitation,’ thus, enabling the model to continue learning while maximizing rewards [60]. Despite potential challenges such as computational intensity and the risk of suboptimal results if not properly balanced, Q-learning proves its worth by aiding in the discovery and optimization of molecular structures and compound characteristics [61]. It particularly excels in multi-property optimization, relationship identification among compounds, and the exploration of molecular space to identify promising candidates.

Further augmenting the capabilities of reinforcement learning in drug discovery is MCTS, another critical tool that enhances decision-making by deftly balancing ‘exploration’ and ‘exploitation’ [62]. Despite its computationally intensive nature and the challenge of striking the right balance, MCTS is indispensable due to its proficiency in navigating vast and complex molecular landscapes. It not only assists in the discovery and design of potential drug candidates but can also customize drugs to bind to specific targets [63,64,65]. MCTS particularly shines in retrosynthetic planning by offering a systematic approach to deconstructing complex organic molecules, thereby streamlining the planning of synthetic routes. By exploring a multitude of synthetic pathways, it helps chemists plan and execute synthesis more efficiently [66]. Additionally, MCTS enhances data mining in drug discovery, unearthing hidden patterns and structures in vast datasets.

### 2.2. Deep Learning Method

A pivotal advancement in the field of AI was the introduction of deep learning (DL), a subset of ML algorithms, designed to mimic the information-processing mechanism of the human brain. The human brain contains approximately 100 billion neurons, the cells that make up the nervous system. These neurons are intricately connected in multiple layers through a structure called synapses, transmitting signals by exchanging electrochemical signals. This structure of the human nervous system inspired the creation of artificial neurons, leading to the concept of ‘perceptrons’, which marked the beginning of artificial neural networks (ANN). A multilayer perceptron (MLP) is a type of neural network that possesses multiple layers, known as hidden layers, situated between the input and output layers. When there are two or more hidden layers, the term ‘deep’ is used, highlighting the use of consecutive layers.

**Figure 3:**
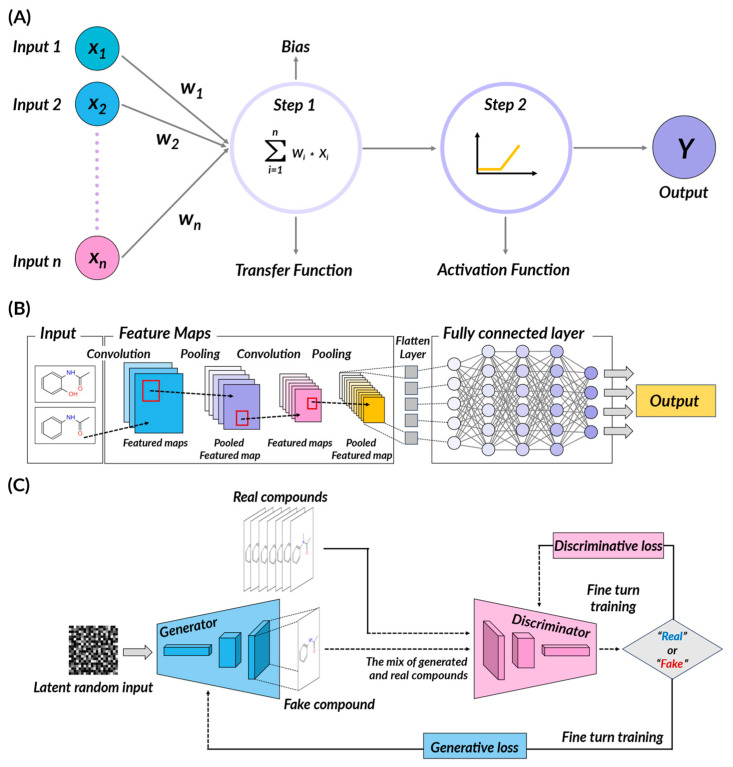
Schematic illustration of deep learning methods. (**A**) Simplified representation of perceptron, a unit component of the artificial neural network (ANN), receiving multiple values as inputs and outputting a single value based on transfer and activation function. (**B**) Schematic diagram of convolutional neural network (CNN). (**C**) Schematic diagram of generative adversarial network (GAN).

In DL algorithms, predicted outcomes are generated through multiple layers using the input data, and this prediction is then compared to the actual value to calculate the difference (Figure 3). To reduce this difference, the weights of the previous layers are adjusted in a process called back-propagation. This process is repeatedly performed to continually refine the model. Examples of popular deep learning algorithms include convolutional neural networks (CNNs), often used in image processing tasks; recurrent neural networks (RNNs), particularly effective for sequence data such as time series or natural language; and deep belief networks (DBNs), which utilize unsupervised learning with generative models. Other examples are autoencoders for creating compact representations and generative adversarial networks (GANs) for generating new data that resemble the input data. These diverse algorithms reflect the breadth and depth of deep learning’s potential applications.

(1) *Convolutional Neural Networks (CNNs)*

Convolutional neural networks (CNNs) utilize small filters in several layers to detect patterns within data. Initially, CNNs extract simple features from the data and then combine them to extract more complex features. CNNs can extract useful features from images of molecular structures in drug discovery. CNNs have the primary advantage of effectively learning complex features from visual data. However, they require large amounts of data and significant computational resources, which can make model results difficult to interpret. Leveraging their fundamental capabilities, CNNs have found expansive usage in drug design, primarily for their proficiency in handling complex, multidimensional data.

In the field of molecular structure analysis, a CNN aids in deriving purposeful features or ‘descriptors’ that represent a compound’s physicochemical properties. For instance, BindScope employs deep convolutional neural networks to classify and visualize compounds on a large scale, based on their activity or inactivity in structure-based drug discovery [67]. Likewise, the use of artificial intelligence (AI) and machine learning technologies, such as a graph convolutional neural network (Graph-CNN), has facilitated the investigation and implementation of diverse molecular representations in drug discovery screening for identifying and predicting inhibitors of SARS-CoV-2 3CLpro [68]. These descriptors allow for accurate prediction of a compound’s behavior, while CNN excels at assessing similarities between different molecular structures, revealing intricate relationships among compounds. For example, in the ligand-based virtual screening approach, the L3D-PLS model employs CNN to extract crucial interaction features from grids surrounding aligned ligands, outperforming traditional methods in the lead optimization of small datasets [69]. In another application, the CAT–CPI model combines CNN with transformers to improve the prediction of compound-protein interactions, accelerating drug development [70]. Additionally, the FRSite method uses a faster R-CNN-based approach to accurately predict protein binding sites, introducing multi-source 3D data and RPN-3D networks to simultaneously predict the center and size of the binding site [71].

Moreover, CNNs are used to group molecular structures based on similarities, which is crucial for comprehending a drug’s impact and improving the performance of ensemble docking by identifying comparable molecular conformations. For instance, a recent study developed a deep learning model for compound classification using a distributed representation of compounds based on the SMILES notation [72]. Using this representation in a convolutional neural network (CNN), the model could process various compound types while obtaining low-dimensional representations of input features and outperforming standard methods in discriminating compound structures, including identified and unidentified motifs. This approach highlights CNNs’ adaptability and effectiveness in medicinal chemistry and enables a more nuanced understanding of the characteristics of compounds and potential drug interactions. Furthermore, in the context of large-scale data mining in drug design, CNNs assist in revealing patterns, correlations, and structures within enormous datasets.

(2) *Recurrent* *Neural Networks (RNNs)*

Recurrent neural networks (RNNs) are a type of neural network designed to process continuous information. Over time, patterns are learned by these networks, which makes them suitable for natural language processing and time-series data analysis. In the case of drug development, RNNs can be beneficial in learning the amino acid sequence of a protein and predicting its impact on a specific illness. The primary benefit of RNNs lies in their ability to understand sequence information. However, as the sequence grows longer, they often fail to retain the initial information, which is a significant drawback.

They aid in constructing innovative drug molecules through various methods, enhancing the field of medicinal chemistry. For instance, combining stack-augmented recurrent neural networks with multi-objective reward-weighted sums in reinforcement learning optimizes the efficient drug design process, proposing a novel way to generate molecules with desired molecular characteristics [73]. By utilizing RNN models, the development of new derivatives of metronidazole and the synthesis and validation of compounds that inhibit bacterial strains such as *E. coli*, *P. aeruginosa*, *B. subtilis*, and *S. aureus* is explained [74]. The application of memory-augmented techniques using RNN-based architectures such as neural Turing machine (NTM) and differentiable neural computer (DNC) in creating new small molecules, analyzing their performance against simple RNNs, and assessing their validity, novelty, and attribute bias in de novo drug design are also explored [63]. These strategies translate chemical attributes into sequences such as simplified molecular-input line-entry system (SMILES), thus, assisting in forecasting novel potential drug candidates. Moreover, the effectiveness of neural networks, including RNNs, in forecasting drug–target interactions has been recognized. For example, a new deep learning approach using graph neural networks based on 3D structural information has been proposed to predict drug–target interactions [75]. The scope of RNN usage further spans the synthesis and testing of drug efficacy, offering a shift from traditional methods to a more data-centric approach, yielding more precise predictions and superior drug candidates.

(3) *Deep Belief Networks (DBNs)*

A deep belief network (DBN) is a type of deep neural network structure that employs multiple restricted Boltzmann machines (RBMs) to stack layers of neurons. DBNs are utilized in drug development as a powerful tool to comprehend complex molecular properties. These learned properties are instrumental in the synthesis of potential drug candidates. DBNs exhibit strength in their capability to learn in an unsupervised manner, which enables them to discern intricate patterns within the input data. However, the down side associated with DBNs is that the learning process is intricate and a demands substantial amount of data and computational resources.

Drawing upon foundational concepts, DBNs demonstrate vast potential in drug discovery, particularly due to their capability to model complex, non-linear relationships in multi-dimensional data. They help to define molecular features and accurately predict the biological activity of novel compounds, thereby aiding in the identification of potential new drug candidates. For instance, AI-driven natural language processing and machine learning algorithms have been applied to explore challenges and opportunities in natural product (NPs) drug discovery, with specific AI approaches developed to identify biologically active natural products and capture the molecular ‘patterns’ of these privileged structures [76]. Notably, DBNs augment data mining in drug discovery, aiding in deciphering patterns, correlations, and structures within large datasets. For example, the application of a deep belief network (DBN) with a dropout mechanism to overcome the overfitting problem associated with small sample sizes has introduced a rapid and non-destructive drug identification method using near-infrared spectroscopy [77].

(4) *Autoencoders*

An autocoder comprises an encoder that compresses input data and a decoder that restores compressed data. In drug discovery, an autoencoder compresses complex molecular properties. These properties are then used to synthesize new drug candidates. Autoencoders have the advantage of learning unsupervised and effectively compressing important characteristics of input data. The disadvantages of autoencoders include their sensitivity to noise in data that contains noise.

Autoencoders have emerged as a powerful tool in drug design, offering promising applications in predicting drug–target binding affinity and generating novel compounds. Specifically, in drug design and synthesis, techniques such as variational autoencoders can be instrumental in engineering novel molecules by decoding latent spaces to generate valid, novel molecular structures that could serve as potential drugs. For example, the problem of generating invalid molecular structures in automated chemical design can be alleviated by recasting it as a constrained Bayesian optimization problem within the latent space of a variational autoencoder, thereby significantly enhancing the validity of the generated molecules [78]. By utilizing their ability to capture and compress high-dimensional data, autoencoders can effectively extract latent features from chemical structures and learn representations that capture the underlying relationships between drugs and their targets. This enables accurate prediction of binding affinities and facilitates the identification of potential drug candidates. For instance, a deep-unsupervised-learning-based method called AutoDTI++ has been proposed to enhance the performance of drug–target interaction (DTI) predictions [79]. Furthermore, autoencoders excel in predicting drug–protein interactions by learning the intricate patterns and dependencies between chemical compounds and protein structures. By encoding the molecular features of drugs and proteins into a lower-dimensional space, autoencoders can effectively capture complex interactions and predict the likelihood of binding events. This capability enhances the performance of virtual screening methods and enables efficient exploration of the vast chemical space. Specifically, a deep learning framework that combines variational autoencoders and attention mechanisms, using CNNs to extract local features, has been proposed to obtain crucial information about drugs and proteins and improve drug–protein interaction (DPI) predictions [80].

(5) *Generative Adversarial Networks (GANs)*

Generative adversarial networks (GANs) comprise a generator and a discriminator, two neural networks that learn from their interactions with each other. GANs can create new data that closely resemble real-world data. In drug development, GANs can produce new drug candidates that resemble real-world drugs. GANs have the advantage of being generative models that can learn from unsupervised data, but the disadvantage is their unstable learning process that makes it challenging to maintain a balance between the generator and discriminator.

Drawing upon their foundational principles, generative adversarial networks (GANs) have seen substantial application in drug discovery, primarily owing to their ability to learn complex data distributions. They play a crucial role in de novo molecular design, wherein deep generative models can effectively learn from existing data and generate novel molecules, addressing the inefficiencies and time-consuming aspects of traditional methods. In particular, recent developments in deep generative models for de novo molecular design have been reviewed, categorizing these models into two types, examining their strengths and weaknesses, and identifying current challenges [9]. Moreover, a new technique utilizing a deep learning GAN called “DNMG” has been proposed to integrate the 3D information of molecules and effectively predict and explore drug properties and binding affinities for new drug design [81].

GANs are also pivotal in the generation and analysis of high-content images in drug discovery assays. Specifically, a computer-based framework that employs three variations of GANs has been proposed for the automatic analysis of large-scale image data generated from drug tests. Among them, the DCGAN, in particular, has been applied to create realistic synthetic images that can be used to study the effects of drugs on cells and bacteria [82]. By learning data distribution, GANs can create synthetic images, enhancing the automatic analysis of voluminous image data generated in drug screening processes. Moreover, they prove instrumental in predicting drug–drug interactions (DDIs) by learning the patterns within large-scale data, leading to an understanding of unforeseen interactions. Specifically, a novel deep learning model known as DGANDDI utilizes two GAN architectures to deeply explore complementary knowledge between drug attributes and DDI network topology [83]. Another significant utilization of GANs is in predicting drug–target interactions, where they can learn the potential interactions between drugs and their respective protein targets, thereby directing the process of drug discovery. In particular, a novel approach using GANs implements a semi-supervised learning method that leverages both labeled and unlabeled data to predict the binding affinity between drugs and targets [84].

### 2.3. Performance Metrics

Performance metrics are tools for evaluating a model’s efficacy, with different metrics utilized for classification and regression tasks. Accuracy, precision, recall, and the F1 score are commonly used to assess how accurately the model categorizes data. On the other hand, for regression, the mean squared error (MSE), mean absolute error (MAE), and root mean squared error (RMSE) measure the discrepancy between predicted and actual values. These metrics play a crucial role in understanding and enhancing model performance.

(1) *Metrics for Classification Models*

Classification models are supervised learning algorithms that categorize given data into one of the predefined classes. Key terminologies for evaluating the performance of the model include true positive (TP), true negative (TN), false positive (FP), and false negative (FN). TP represents the number of positive cases correctly detected by the model, making it an important metric for evaluating model performance. TN represents the number of negative cases correctly ignored by the model, which is especially crucial when wanting to avoid incorrect positive predictions. FP is when the model incorrectly predicts a data point that is actually negative as positive. Such predictions can give users incorrect information or unnecessary alerts. FN is when the model incorrectly predicts a data point that is actually positive as negative. FNs are missed positive cases by the model, which can have severe consequences. For instance, in a medical diagnostic model detecting diseases, an FN could result in a patient with the disease not receiving a diagnosis. Such terminology forms the basis for calculating performance metrics of classification models, which are essential to accurately determine the strengths, weaknesses, and areas of improvement of the model. Based on these terms, several performance metrics are calculated. These metrics reflect various aspects of classification model performance such as accuracy, sensitivity, and specificity, and their importance can vary depending on the specific application area [85].

The *accuracy* (*ACC*) indicates the proportion of data correctly classified by the model among its predictions. It is useful when the class distribution of the data is uniform. If one class greatly outnumbers the other, it can be challenging to fully assess the model’s performance based solely on accuracy.

$$ACC=\frac{\#correctly\;classified\;samples}{\#\;All\;samples}=\frac{TP+TN}{TP+FP+TN+FN}$$

The *precision* (*PREC*) denotes the ratio of data points that are actually positive among those the model classified as positive. This metric becomes important when the cost of incorrect positive predictions is high.

$$PREC=\frac{\#samples\;correctly\;classified}{\#\;samples\;assigned\;to\;class}=\frac{TC}{TC+FP}$$

The *recall* (*REC*) or *sensitivity* represents the ratio of data points the model predicted as positive among the actual positive data points. It is critical when the cost of incorrect negative predictions is high.

$$REC=\frac{\#true\;positive\;samples}{\#\;samples\;classified\;positive}=\frac{TP}{TP+FN}$$

The *F*1 *score* is the harmonic mean of precision and recall, indicating a balance between the two metrics. It is especially useful when one class’s sample size is much smaller than the other.

$$F1=2\times \frac{precision\times recall}{precision+recall}=\frac{2\times TP}{2\times TP+FP+FN}$$

The *specificity* (*SPEC*) illustrates the ratio of actual negative class data points correctly predicted as negative by the model.

$$SPEC=\frac{\#true\;negative\;samples}{\#samples\;classified\;negative}=\frac{TN}{TN+FP}$$

All these metrics fall within the [0, 1] range, where 1 indicates perfect prediction based on the metric, and 0 indicates an entirely incorrect prediction. Each performance metric emphasizes different aspects of the model, so the importance of a particular metric can increase depending on the specific application or problem. Therefore, it is crucial to consider these metrics comprehensively when evaluating and optimizing model performance.

(2) *Metrics for Regression Models*

A regression model is an algorithm that models the relationship between one or more independent variables and a continuous dependent variable. Such models are used to predict a continuous output value for given input variables. Various metrics are employed to assess the performance of a regression model by measuring the difference between the predicted and actual values.

The *mean squared error* (*MSE*) is the average of the squared differences between predicted and actual values. Due to its squaring of prediction errors, large errors can significantly inflate this value.

$$MSE=\frac{1}{n}\overset{n}{\underset{i=1}{\sum }}{\left(y_{i}-{\tilde{y}}_{i}\right)}^{2}$$

The *mean absolute error* (*MAE*) is the average of the absolute differences between predicted and actual values. It calculates the absolute error for each prediction and then averages those values.

$$MAE=\frac{1}{n}\overset{n}{\underset{i=1}{\sum }}\left|x_{i}-x\right|$$

The *root mean squared error (RMSE*) is the positive square root of MSE. RMSE provides an interpretation of the prediction error size in the original unit. While it gives greater weight to large errors, RMSE can be more interpretable as it represents the actual size of the prediction error.

$$RMSE=\sqrt{\frac{1}{n}\overset{n}{\underset{i=1}{\sum }}{\left(y_{i}-{\tilde{y}}_{i}\right)}^{2}}$$

The *R-squared*, also known as the coefficient of determination, is a metric that illustrates the predictive power of a regression model. It indicates how well the model explains the variability in the data.

$$\begin{array}{c} R^{2}=1-\frac{\sum {\left(y_{i}-{\tilde{y}}_{i}\right)}^{2}}{\sum {\left(y_{i}-{\overset{ˇ}{y}}_{i}\right)}^{2}}\;\\ \tilde{y}=predicted\;value\;of\;y\\ \overset{ˇ}{y}=mean\;value\;of\;y \end{array}$$

These metrics play a crucial role in evaluating the performance of a model, emphasizing different aspects. Therefore, when assessing and optimizing the performance of a regression model, it is essential to consider these metrics comprehensively.

### 2.4. Databases in Drug Research

The effectiveness of ML or DL methods is predominantly contingent upon the availability of substantial, accurate, and reliable data. As technological advances continue to make data generation faster and more affordable, the volume of chemical, biological, and medical data has grown exponentially. There is a continuous effort to centralize these datasets and make them publicly available for research around the globe. This crucial information can be procured from many public databases, which serve as rich repositories of information that are indispensable for drug discovery. These databases encapsulate a wide array of knowledge about the efficacy of various drugs, potential side effects, the nature of drug targets, and intricate chemical structures. However, it is essential to understand that not all databases are equivalent in terms of the type and depth of information they contain. Each database is unique, housing specific sets of data. Therefore, gaining a robust understanding of the nature of the information these databases provide, and how to utilize it effectively, is essential for researchers. Table 1 summarizes the publicly available databases that contain various aspects of data utilized in drug discovery.

## 3. AI in Structural Biology

Structural biology, encompassing the study of the three-dimensional (3D) structures of biological macromolecules, is essential for understanding the precise mechanisms underlying living organisms. Experimental techniques such as X-ray crystallography, nuclear magnetic resonance (NMR) spectroscopy, and cryo-electron microscopy (cryo-EM) have been employed to elucidate numerous structures. However, these experimental methods often require extensive labor, time, and financial resources, posing limitations for specific molecules or complexes. AI has advanced structural biology by providing computational approaches to overcome these experimental limitations [118,119]. AI techniques, especially DL methods, and data-driven modeling, have been applied by extracting meaningful patterns and features from vast and complex biological sequence and structural data. AI offers innovative tools for researchers, enabling protein structure prediction, protein design, and more, and continues to evolve alongside advancements in hardware and algorithm.

### 3.1. Protein Folding and Its Prediction

Protein folding, a highly intricate process, plays a fundamental role in determining the functional properties of biological macromolecules. Despite the limited number of around 20 amino acids, the varying structures and functions depending on their arrangements are of significant research interest in life sciences. Therefore, structural studies on protein folding are essential for understanding biological mechanisms and developing therapeutic strategies for diseases. However, observing the actual structure of proteins requires substantial resources and time. Hence, researchers attempt to predict protein folding and 3D structures using genetic information.

Despite the high demand for protein structure prediction, it remains challenging, and achieving perfect prediction is still elusive. Even minor variations in protein sequences can lead to drastic changes in overall structure and, in some cases, result in loss of function. On the other hand, certain amino acids share similar chemical properties, which can lead to minimal structural differences in some mutations. Additionally, despite the astronomical range of conformational possibilities resulting from the rotations of amino acids in flexible polypeptide chains, most small proteins fold spontaneously on a millisecond or even microsecond timescale. In order to address these Levinthal’s paradoxes, scientists have conducted extensive research in this area [120,121]. Researchers have attempted to predict structures using computational thermodynamic hypotheses, but this method has not yielded perfect results. For instance, predicting conformations of a protein within a biological system is challenging because even slight inaccuracies in the computation of the significant free energy difference between folded and unfolded states can lead to incorrect predictions. Therefore, recent arguments suggest that instead of traditional thermodynamic hypotheses, the non-equilibrium and active nature of proteins within the biological context requires modeling using fluctuating free-energy landscapes [122].

To overcome these limitations, various advancements and refinements have been made in the field of homology modeling techniques. Homology modeling, also known as comparative modeling, predicts the structure of a target protein using experimentally determined structures of homologous proteins as templates. Homology modeling contributes to understanding protein structure and function, aiding hypothesis generation and experimental design. This approach leverages the principle that proteins with similar sequences adopt similar structures. Key steps include selecting a suitable template with high sequence similarity, aligning the target sequence with the template, generating a model, and model optimization followed by validation [123]. The model accuracy largely depends on sequence similarity, and adopting strategies such as utilizing multiple templates, along with implementing processes such as energy minimization and loop modeling, can significantly enhance this precision.

### 3.2. Biomolecular Structure Prediction by Computational Methods

As research methods and technologies have advanced, protein structure and sequencing data accumulation has been accelerating. The Protein Data Bank (PDB), established in 1971, serves as the primary archive housing the largest collection of protein 3D structures [124]. As of 2022, the number of experimentally determined 3D protein structures has exceeded 200,000, with the pace of data accumulation steadily increasing [125]. Additionally, the UniProt Knowledgebase (UniProtKB) [109] provides information on protein sequences including functional annotations and currently holds over 220 million sequences as of 2023. This database also encompasses protein structure visualization data, including predicted structures by AI (i.e., AlphaFold, which is described in the following section).

MODELLER [126], which started development in 1993, is a representative program that can generate homology models using these accumulated databases. When researchers provide the sequence of the desired protein and structures of similar sequences, the program automatically creates models that satisfy spatial restraints using comparative protein structure modeling [127]. The SWISS-MODEL server, another tool in the field, provides an automated web service for generating homology models, with the ProMod3 modeling engine at its core [128]. This program utilizes QMEANDisCo [129] for model quality estimation and has demonstrated excellence through the CAMEO project, showcasing its effectiveness [130]. I-Tasser [131] has garnered significant attention due to an outstanding performance. I-Tasser combines various methods, including threading, ab initio modeling, and structure assembly, to generate models [132]. It utilizes a hierarchical approach, starting with the identification of template structures through threading, followed by fragment assembly simulations and refinement. The program has demonstrated competitive performance in several international structure prediction competitions, such as critical assessment of protein structure prediction (CASP) [133].

**Figure 4:**
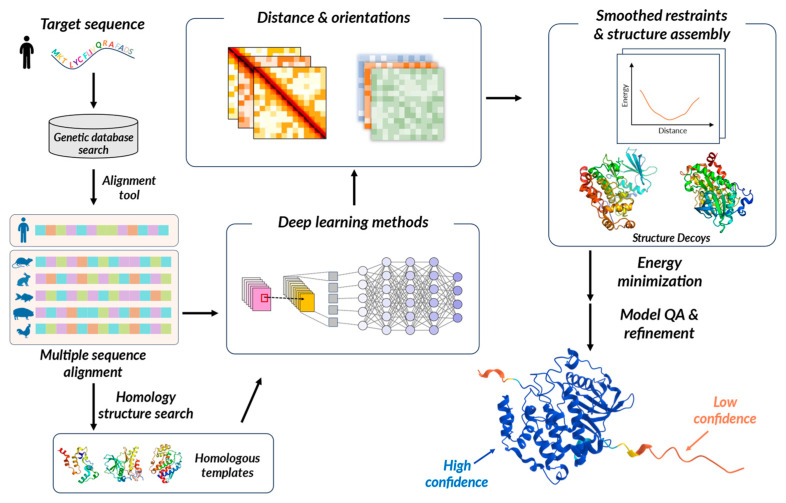
Workflow of DL-based protein structure prediction.

Collaborative efforts such as CASP are underway, providing a valuable platform for evaluating AI-based biomolecule structure prediction research [134,135]. Various research teams engage in structure prediction by categorizing protein sequences into cases amenable to template-based modeling (TBM) or those requiring free modeling (FM) and then evaluating their predictions. In 2018, DeepMind’s AlphaFold participated in CASP13 and introduced a novel approach by enhancing the traditional fragment assembly technique using deep learning (DL) methods. AlphaFold utilizes a deep residual convolutional neural network (CNN) to effectively capture intricate patterns within the protein data (Figure 4). The neural network undergoes training to make predictions about protein structures, and through gradient descent, it minimizes the potential energy to stabilize and achieve accurate structure prediction [136]. AlphaFold2 emerged during CASP14, introducing a novel neural network block called Evoformer [119,135]. Evoformer enhances the accuracy of structure prediction by facilitating the exchange of information within the multiple sequence alignment (MSA) and learning the relationships between sequences. It captures intricate spatial relationships, thereby improving the prediction of protein structures. trRosetta is also an approach that incorporates CNN into the existing RosettaFold framework [137]. By utilizing CNN, it directly predicts inter-residue distances and torsion angles from protein sequences and MSAs. These predictions are then integrated with the fragment assembly approach of RosettaFold to generate protein structure models.

### 3.3. Advancements in Protein Structural Research through AI

With the successful application of AI in predicting the structures of individual proteins, there has been a growing interest in applying AI to a broader range of structural biology research. One such area is the prediction of protein complex structures, which is expected to make significant contributions to the study of host–pathogen interactions [118]. DeepMind has released AlphaFold-Multimer to predict protein complex structures [138,139]. However, the coevolution within a protein and between proteins exhibits distinct patterns, posing limitations to complex prediction using MSA-based methods. Moreover, there are challenges in accurately predicting heterodimeric complexes compared to homodimeric ones, and the accuracy tends to decrease as the number of chains increases. In response to these issues, efforts have been made to address them by introducing ESMFold [140], a language model-based approach, aimed at improving the prediction accuracy.

The epigenetic dimension of protein structure (EDPS) represents another important objective that needs to be addressed [141]. Currently, neural network (NN)-based modeling algorithms face significant limitations in epigenetic protein structure prediction. This is particularly evident in membrane proteins [142], where template-based modeling (TBM) demonstrates higher accuracy than NN-based modeling, owing to the incorporation of lipid bilayer template data. This observation suggests that specific lipid species in the membrane environment may influence protein structure formation [143,144]. Comparative studies on G-protein-coupled receptor (GPCR) structure prediction supports this notion [145], as TBM exhibits relatively higher accuracy in loop regions, possibly due to the influence of the surrounding environment. Consequently, further research is required to explore additional methods that can improve the accuracy of EDPS prediction, especially for membrane proteins, even in the absence of suitable templates.

The integration of AI in protein structural research has shown notable advancements and potential in this field. While some challenges persist, particularly in predicting complex interactions and navigating the epigenetic aspects of protein structure, AI’s adaptability and the ongoing refinement of its methodologies have shown promise in overcoming these hurdles. Tools such as AlphaFold or ESMFold represent the innovative solutions developed to address existing limitations. However, the journey towards the comprehensive and accurate prediction of protein structures, including complex and epigenetically influenced structures, is ongoing. As AI technologies continue to evolve, it is anticipated that they will play an increasingly critical role in unfolding the mysteries of protein structures, thereby revolutionizing our approach to structure-based drug discovery.

## 4. AI in Medicinal Chemistry or Cheminformatics

AI has also revolutionized medicinal chemistry and cheminformatics by providing innovative tools and approaches for drug discovery, such as deep generative models for molecular design, as well as the prediction of drug–target interactions or drug toxicity [47]. AI-driven approaches enable the exploration of vast chemical space, leading to the discovery of novel compounds with therapeutic potential. They can also facilitate drug repurposing by analyzing large-scale data to identify connections between drugs and diseases, expanding the possibilities for addressing unmet medical needs. Regarding chemical structures, AI methods generally use molecular fingerprints as input data. They are trained to find patterns in these fingerprints that correlate with the properties of interest, such as biological activity or physicochemical properties. This process is crucial in various cheminformatics tasks such as virtual screening, QSAR modeling, and de novo drug design. Thus, the relationship between molecular fingerprints and AI methods in cheminformatics is one of symbiosis, with each enabling the other’s functionality.

### 4.1. Molecular Fingerprints

Molecular fingerprints are binary or count-based representations that encode the chemical structure into a format that computational models can readily process. They can be seen as a bit string (usually several hundreds or thousands of bits long), where each bit represents the presence or absence of a particular substructure or property within the molecule [146]. Capturing different aspects of the molecular structure, such as the presence of certain functional groups, topological features, or 3D properties, it can transform these complex structures into simplified, standardized vectors [147]. The primary use of the molecular fingerprint is for creating a simplified digital representation of complex molecules, which allows efficient comparisons and searches in large molecular databases. It can be also used as a particular form of molecular descriptor that is commonly applied to a wide range of tasks, from virtual screening to prediction model building [148].

**Figure 5:**
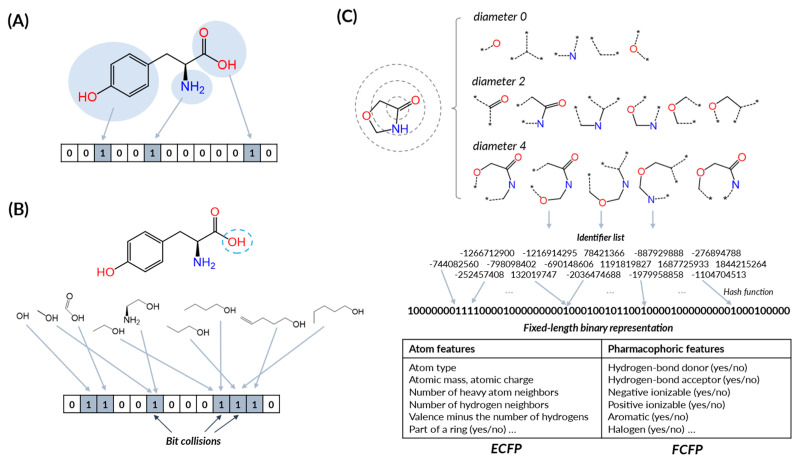
Schematic representation of molecular fingerprints. (**A**) Substructure key-based method. (**B**) Topological or path-based method. (**C**) Circular methods.

Molecular fingerprints can be categorized into the following classes [149,150,151]: (1) *substructure keys-based fingerprints* that generate bit strings depending on the presence or absence of substructures in the compound, (2) *topological fingerprints* that focus on topological routes and represent all possible connectivity routes between atoms, (3) *circular fingerprints* that are also related to atom paths similar to topological fingerprints but represent the connectivity of atoms not in a linear way but in a radius way, illustrating the atom’s environment [152], and (4) *pharmacophore fingerprints* that encode atoms by the pharmacophoric functional groups and atom bonds by several distance ranges [153]. Some fingerprints cannot be in these classes. SMIfp [154], which is calculated from SMILES, is a good example of that exception. Figure 5 and Table 2 summarizes the representative fingerprints commonly used in drug discovery research.

Fingerprints can be used to calculate molecular similarity or distance and help identify molecules with similar structures or properties, clustering molecules into groups, or predict the properties or activities of new molecules based on their similarity to known ones. As a tool in machine learning, molecular fingerprints can be used as input features for models predicting the properties or activities of molecules. In this context, the model learns to associate certain patterns in the fingerprint with the property or activity being predicted. The major drawback of molecular fingerprints is that they generally ignore biological context and do not contain 3D information. Various approaches have been proposed to overcome these limitations. For example, atom pair 3D fingerprints are developed from 2D atom pair fingerprints to contain 3D information [155]. Protein–ligand interaction fingerprints (PLIF) encode information about protein–ligand interactions, such as hydrogen bonds, ionic interactions, and surface contacts according to the residues [156].

**Table 2:** Various types of molecular fingerprints.

Category	Molecular Fingerprint	Description	Ref.
Substructure key-based	Molecular ACCess system (MACCS) keys	▪ The most commonly used structural fingerprint, often referred to as the MDL keys ▪ Each bit is associated with a SMILES arbitrary target specification (SMARTS) pattern. ▪ Length: 166 key bits (open source)	[157]
	PubChem fingerprint	▪ Substructure-based fingerprint specifically designed for the PubChem DB (can be retrieved by PubChemPy in python) ▪ Each bit represents a particular substructural feature classified into seven sections written in SMARTS and SMILES. ▪ Length: 881 bits	[158]
Topological	Daylight fingerprint	▪ Hashed topological fingerprint based on the connectivity of atoms in the molecule ▪ Length: variable lengths up to 2048 bits	[150]
	Atom pairs2D fingerprints (APFP)	▪ An atom pair substructure is defined as a triplet of two (non-hydrogen) atoms and their shortest path distance in the molecular graph, i.e., (atom type 1, atom type 2, geodesic distance) ▪ Based on topological routes ▪ Length: variable lengths	[159]
Circular	Extended-connectivity fingerprints (ECFP)	▪ Circular fingerprint based on the Morgan algorithm ▪ Iterative (“extended”) assignment of unique identifiers to atoms based on their local environment (i.e., the atoms they are connected to) ▪ Length: variable lengths	[152]
	Molprint2D	▪ Circular fingerprint where each atom is represented by its local environment up to a defined number of bonds, similar to ECFP ▪ Count-based method (instead of binary type) containing information on heavy atom types and hybridization states ▪ Lengths: variable length up to 2^50^	[160]
Pharmacophoric	Functional-class fingerprints (FCFP)	▪ A variant of extended-connectivity fingerprint (ECFP) ▪ While ECFPs focus on atom connectivity to create fingerprints, FCFPs incorporate information about the functional roles of atoms in the molecule ▪ Length: variable lengths	[152]
SMILES-based	SMIfp	▪ Based on the number of occurrences of symbols found in the SMILES ▪ Length: variable lengths	[154]

### 4.2. Deep Generative Model for Molecular Design

A deep generative model is a type of neural network trained on existing data with a high-dimensional probability distribution, which it then uses to create new samples from that distribution [10]. In drug discovery research, deep generative models can be utilized in the area of de novo molecular design, a computational methodology that generates novel molecules with desired properties [8,161,162]. While QSAR models can be used to predict the biological activities of unknown chemicals based on their structures, which are derived from other chemicals with various known biological activities, de novo design is employed to generate novel chemical structures with desired pharmacological properties, using structure–bioactivity data as a basis [161]. They are trained on large databases of known molecules to learn the underlying patterns and structures in the data. This learned knowledge is then leveraged to generate new, unseen molecules that are likely to be physicochemically promising or biologically active.

Popular DL algorithms, such as recurrent neural networks (RNNs), variational autoencoders (VAEs), and generative adversarial networks (GANs), have been applied to de novo molecular design. RNNs are commonly used for data sequence modeling and generation and sequence-to-sequence mapping. In de novo design, they can also be used for analyzing molecular sequence data, such as SMILES [162]. Once the RNNs are trained by target sequences, they can generate new sequences that follow the conditional probability distributions learned from the training set [162]. VAEs can represent high-dimensional complex data by learning a low-dimensional latent space in an unsupervised manner [163]. When the VAE model is applied to de novo design, the encoder converts the molecule to a latent vector representation, and the decoder generates a novel chemical space from the latent vector representation [164]. GANs contain a generator and a discriminator. The generator creates new data based on input data, and the discriminator identifies whether the data are real or generated. During the training, these two components compete against each other, and when the discriminator cannot distinguish between generated data and real input data, the training ends [164]. In this process, GANs can generate novel molecules by using patterns and structures in a pre-existing training dataset.

Deep generative models, despite facing certain challenges, have shown remarkable potential for de novo design in early discovery stages. These models can be utilized to generate novel and diverse chemical scaffolds with desirable predicted values. However, to fully realize their potential, further research is needed to address key issues, such as lack of interpretability. Nonetheless, the early successes of these models have opened up exciting avenues for innovation and creativity that were previously unattainable.

### 4.3. Prediction of Drug–Target Interaction (DTI)

Drugs control our body’s physiological activities to exert therapeutic effects, which are achieved through the interaction between the drug and its target protein, known as drug–target interaction (DTI) [165]. A drug, which serves as a ligand, binds to the pocket in a protein, often referred to as a binding site, inducing changes in physiological activity. These pockets can vary in size and depth, and ligand binding can alter the protein structure, impacting its function through molecular interactions such as ionic bonds, van der Waals interactions, and hydrogen bonds [166,167,168]. They can prevent the protein from interacting with endogenous molecules or cause changes in its activity [165]. This activity can be affected not only by the ligand itself but also by water molecules, metal ion coordination, and various other circumstances [166].

Traditionally, the drug discovery approach has been based on the “one molecule–one target–one disease” paradigm, where the drug produces therapeutic effects by regulating its target. In this approach, it is necessary to test whether a particular protein could be a specific drug target for treatment [169]. However, a single target is not exclusively associated with one disease, and the onset of complex diseases may involve multiple factors. Depending on the circumstances, it may be necessary to intervene in multiple areas along the pathologic mechanism for effective treatment [170]. From this perspective, the importance of DTI research is increasingly recognized, especially regarding side effects, drug repositioning, and drug resistance [171].

The power of AI is apparent in this target identification and virtual screening [172]. ML algorithms, especially during the virtual screening process, follow an approach that differs from conventional structure-based virtual screening (SBVS) or ligand-based virtual screening (LBVS) [35]. They generate statistical models to anticipate the conditions of undiscovered ligands–proteins based on the recognized configurations of protein–ligand compounds and physicochemical characteristics [166]. The research carried out on the three aspects of ML-based DTIs, namely, prediction of existing ligand binding sites, binding affinity, and binding poses, is ultimately aimed at bringing us closer to more efficient drug discovery [170]. Table 3 summarizes a recent application of AI in DTI prediction.

### 4.4. Toxicity Prediction

The significant increase in chemical usage has intensified the need for reliable toxicity prediction, leading to the establishment of the Tox21 program in 2008, a collaborative endeavor undertaken by the U.S. governments, such as the Environmental Protection Agency (EPA). Further strengthening this initiative, the U.S. Food and Drug Administration (FDA) became a part of the consortium in 2010. This program employs high-throughput screening (HTS), an in vitro assay, to scrutinize the biochemical activity of various substances. This methodology has the advantage of not only reducing the time and cost associated with toxicity testing but also mitigating ethical concerns [187,188]. The culmination of these efforts resulted in the creation of the Tox21 10K library, which subsequently fostered the inception of the Tox21 data challenge, an initiative designed to enhance the precision of predictions about holistic human responses utilizing computational methodologies [189]. In parallel, EPA’s Toxicity Forecaster (ToxCast) program, instituted in 2007, assesses materials that could be harmful to human health and the environment via bioactivity profiling [190]. Distinguished from Tox21, ToxCast covers a broader chemical space and grapples with less specific mechanisms of action. Implementing methods analogous to HTS, it endeavors to categorize chemicals and advocate for the appropriate regulation of environmental pollutants [191]. These initiatives persist as critical contributions to the progression of toxicity prediction models.

These valuable Tox21 and ToxCast datasets have been monumental in driving advancements in the field of ADME/Tox prediction. Compounds that enter the human body commonly undergo absorption, distribution, metabolism, and excretion (ADME), with some leading to toxicity [192]. Efforts to decode these processes from a pharmacokinetic/pharmacodynamics (PK/PD) perspective have been put forth but given their interactions with numerous human body structures such as membranes, proteins, and the intra/extracellular environment, the ADME–Tox (ADMET) processes are viewed as multifactorial and intricate. Factors such as the compound’s solubility, membrane permeability, consumed concentration, and partition coefficient play a significant role in the absorption process [193,194]. AI algorithms can be trained on the Tox21/ToxCast datasets to predict the potentially toxic effects of a new compound, which are critical aspects of the drug discovery process [195]. Here, we describe recent applications in three representative endpoints, i.e., hepatotoxicity, cardiotoxicity, and carcinogenicity.

(1) *Prediction of Hepatotoxicity*

Given the crucial role the liver plays in drug metabolism, assessing the potential for drug-induced liver injury (DILI) is vital for safety reasons [196,197]. Hepatotoxicity, in particular, is a significant issue in drug development, leading to a significant number of drugs being withdrawn due to DILI or not being launched at all [198,199]. Safety concerns mean that only about 31.8% of potential drug candidates progress from preclinical testing to clinical trials [200]. Even among the drugs that are successfully launched, 58% of FDA drugs approved in 2020 and 2021 show signs of hepatotoxicity [201]. Traditional methods of DILI evaluation, such as in vitro and in vivo studies, are both costly and time-consuming [202]. In response to this, there has been a shift towards developing computational methods that can predict DILI quickly and accurately using AI methods.

(2) *Prediction of Cardiotoxicity*

Cardiotoxicity is a paramount concern in the creation of innovative pharmaceuticals [203]. In line with the International Conference of Harmonization’s guideline (S7B), it is obligatory for all emergent drugs to undergo a pre-clinical examination of their potential to inhibit hERG activities before they are considered for regulatory appraisals [31,204]. The hERG channel, alternatively known as Ether-à-go-go (EAG) proteins, constitutes potassium channels that are manifested in diverse brain areas, endocrine cells, muscles, and the heart [205,206]. These channels play an indispensable role in cardiac function by facilitating the heart’s electrical activity [205]. The blockade of these channels by small molecules can precipitate QT interval prolongation, which may culminate in lethal cardiotoxicity [207,208]. Consequently, it is essential that drug candidates demonstrate minimal hERG inhibition to circumvent such deleterious effects [209].

(3) *Prediction of Mutagenicity and Carcinogenicity*

Mutagenicity and carcinogenicity are key considerations in the risk assessment of chemicals and pharmaceuticals [210,211]. Mutagenicity refers to a substance’s ability to cause genetic mutations, potentially leading to various disorders, including cancer, while carcinogenicity is a compound’s potential to cause cancer [212,213,214]. Given the correlation between these two and the global burden of cancer, it is vital to evaluate mutagenicity and carcinogenicity [215,216,217]. Challenges in this task include mutagenicity, inconsistencies in Ames test results, false positives and negatives, and reproducibility issues among labs [218,219]. The 1995 ICH guidelines provided a structure for carcinogenicity studies, yet these studies require two years, approximately $1.1 million, and about 500 rodents, making it a laborious and costly process [220]. As such, in silico predictive methods are gaining popularity, with several proposals suggesting the use of ML approaches to increase efficiency [221]. Table 4 summarizes the additional case studies for predicting various toxicity.

## 5. Conclusions and Future Perspectives

In conclusion, the integration of AI in drug discovery represents a significant paradigm shift in medicinal chemistry, rather than just a technological addition. AI unlocks insights from complex datasets that were previously unreachable, and its value in data-driven drug discovery is poised to become increasingly prominent. However, alongside the high expectations for AI’s potential, it is vital to exercise caution. AI models heavily rely on large amounts of high-quality data, making access to diverse and sufficient data crucial for accurate learning and prediction. In the context of drug discovery, these data encompass information about known compounds, biological processes, disease mechanisms, clinical data, patient adverse events, and more. Due to the intricate nature of biological processes, the multitude of variables impacting drug action, and individual variations, many aspects cannot be interpreted or applied in isolation. This complexity presents challenges for achieving full automation, often requiring domain knowledge-based optimization and expert-guided manual curation. Efforts to address these limitations of AI applications have gained momentum across multiple domains. A significant emphasis is now on gathering or digitalizing diverse datasets, ensuring that AI tools represent a comprehensive spectrum of the data. To overcome data deficiency, researchers are using pre-trained models and fine-tuning them on specific, smaller datasets. This approach makes AI applications more adaptable and data efficient. There is also a concerted effort to bolster the robustness of AI, certifying that it performs reliably on previously unseen data.

Drug discovery and development, especially within the life-critical industry, necessitate human involvement for real-world experimental validation and clinical trials, extending beyond virtual simulations alone. Incorporating AI into these fields amplifies some of the ethical considerations, especially around data privacy, transparency, or potential bias. Furthermore, using AI on electronic medical records could risk patient privacy breaches. Nevertheless, AI technology can greatly enhance the efficiency of experiments for researchers, particularly in computationally intensive tasks or in identifying intricate patterns that might evade human observation. The optimal scenario is one where humans and AI technology collaborate, each leveraging their respective strengths. Addressing technical challenges, ensuring data robustness, validating AI models, and considering ethical implications requires a continuous collaborative approach involving academia, industry, and regulatory agencies.

## Figures and Tables

**Table 1:** Databases utilized in drug discovery research.

Database	URL *	Description	Ref.
***Compound and Drug Databases***			
**PubChem**	https://pubchem.ncbi.nlm.nih.gov/	Launched in 2004 as part of the Molecular Libraries Roadmap Initiatives by the US National Institutes of Health (NIH), PubChem is a public database for information regarding chemical substances and their biological activities.	[86]
**ChEMBL**	https://www.ebi.ac.uk/chembl/	ChEMBL is a well-curated database of bioactive molecules with drug-like properties, integrating chemical, bioactivity, and genomic data to aid in the transformation of genomic information into effective new drugs.	[87]
**ZINC**	https://zinc.docking.org/	ZINC is a free database of over 230 million commercially available compounds in 3D formats, suitable for virtual screening, provided by the Irwin and Shoichet Lab at UCSF.	[88]
**ChemSpider**	http://www.chemspider.com	ChemSpider is a free-access website that serves as a chemical database and a structure-centric community for chemists, aiming to aggregate and index accessible information on chemical structures and related data from various online sources. It includes analytical data, synthesis reactions, experimental properties, and more.	[89]
**DrugBank**	http://www.drugbank.ca	DrugBank is a robust online database that provides wide-ranging biochemical and pharmacological data about drugs, including their mechanisms of action and targets.	[90]
**DrugCentral**	http://drugcentral.org/	DrugCentral is a publicly accessible online compendium that consolidates information on the structure, bioactivity, regulatory, and pharmacological actions, and indications of active pharmaceutical ingredients approved by the FDA and other regulatory bodies.	[91]
**Drugs@FDA**	https://www.accessdata.fda.gov/scripts/cder/daf/	Drugs@FDA is a comprehensive database that contains information about FDA-approved prescription and over-the-counter drug products, including brand-name and generic drugs, as well as many therapeutic biological products, with majority of data dating back to 1998 and some extending to 1939.	[92]
***Metabolic and Biomolecular Pathway Databases***			
**KEGG**	https://www.kegg.jp	KEGG is a database designed to provide insights into the high-level biological functions of cells, organisms, and ecosystems using molecular-level data, particularly from large-scale genome sequencing and other high-throughput experiments.	[93]
**BioCyc**	https://biocyc.org/	BioCyc is a comprehensive collection of pathway/genome databases and a suite of bioinformatics tools that offer insights into the genomes, metabolic pathways, and regulatory networks of numerous sequenced organisms, helping to accelerate scientific research.	[94]
**Reactome**	https://reactome.org	Reactome is an open-access, peer-reviewed pathway database that aims to offer user-friendly bioinformatics resources for visualizing, interpreting, and analyzing pathway information. These resources aid various fields, including basic research, genome examination, modeling, systems biology, and education.	[95]
**HMDB**	http://www.hmdb.ca	The Human Metabolome Database (HMDB) is an open-access online database that provides comprehensive information regarding small molecule metabolites identified in the human body.	[96]
***Protein–Protein Interaction and Network Databases***			
**IntAct**	http://www.ebi.ac.uk/intact/	IntAct is a freely accessible database that houses molecular interaction data, obtained either directly from data submissions or curated from scholarly publications.	[97]
**BioGRID**	https://thebiogrid.org	BioGRID is an online repository that meticulously compiles and hosts extensive data on protein and genetic interactions, chemical associations, and post-translational modifications from major model organisms.	[98]
**STRING**	https://string-db.org/	STRING is a comprehensive repository that comprises both acknowledged and projected protein associations. These interactions encompass both direct interactions, which involve physical contact, and indirect ones, which imply functional relationships.	[99]
**STITCH**	http://stitch.embl.de/	STITCH is a platform used to investigate established and anticipated connections between proteins and chemicals, with connections supported by experimental data, databases, and the academic literature.	[100]
***Drug–Target Interaction Databases***			
**BindingDB**	http://www.bindingdb.org/bind/index.jsp	BindingDB is an open, web-based database dedicated primarily to measuring binding affinities between proteins, viewed as drug targets, and small, drug-like molecules.	[101]
**TTD**	http://db.idrblab.net/ttd/	The Therapeutic Target Database (TTD) is a resource that offers details regarding established and potential therapeutic protein and nucleic acid targets, the diseases they target, associated pathway information, and the specific drugs designed to interact with these targets.	[102]
**IUPHAR/BPS Guide to PHARMACOLOGY**	https://www.guidetopharmacology.org/	The IUPHAR/BPS Guide to PHARMACOLOGY is an expert-curated database offering comprehensive information on drug targets, prescription medicines, and experimental drugs, enriched with links to other databases, aiming to be a centralized resource for pharmacology and drug discovery.	[103]
**DGIdb**	http://www.dgidb.org	The Drug–Gene Interaction Database is an online tool that amalgamates various datasets detailing interactions between drugs and genes, and the druggability of genes. It presents a user-friendly visual interface and a well-documented API for data queries.	[104]
***Toxicity and Side Effect Databases***			
**CTD**	http://ctdbase.org/	CTD is a comprehensive, public database that collates data from various sources on the impacts of environmental exposures on human health, including chemical genes, chemical disease, and chemical–exposure interactions across all species, offering analytical tools for hypothesis generation.	[105]
**DrugMatrix/ToxFX**	https://ntp.niehs.nih.gov/data/drugmatrix	DrugMatrix, accompanied by its reporting system ToxFX, serves as one of the largest toxicogenomic reference databases, providing comprehensive profiles for over 600 compounds, aimed at enhancing the efficiency of toxicological assessments and understanding of the potential toxicity of xenobiotics.	[106]
**OECD eChemPortal**	https://www.echemportal.org/echemportal/	eChemPortal is a free public global database that collects and provides direct links to chemical characteristics data and safety information from various national, regional, and international government programs.	[107]
**SIDER**	http://sideeffects.embl.de/	SIDER is a database that provides information about marketed drugs and their documented adverse reactions, including side effect frequency, drug classifications, and additional resources such as drug–target relations.	[108]
***Protein and Gene Databases***			
**UniProt**	https://www.uniprot.org	UniProt offers the scientific community a thorough, superior, and freely accessible database of protein sequences and functional data.	[109]
**InterPro**	https://www.ebi.ac.uk/interpro/	InterPro facilitates the functional examination of proteins by grouping them into families and forecasting the presence of domains and significant sites.	[110]
**GenBank**	http://www.ncbi.nlm.nih.gov/genbank/	GenBank is the NIH’s genetic sequence database, a comprehensive, annotated collection of all publicly accessible DNA sequences, participating in the International Nucleotide Sequence Database Collaboration, with data updates every two months.	[111]
**RCSB PDB**	http://rcsb.org/	RCSB PDB is a resource-driven by the Protein Data Bank archive, offering detailed information about 3D structures of proteins, nucleic acids, and complex assemblies, aiding students and researchers in exploring biomedicine, agriculture, protein synthesis, and various health and disease conditions.	[112]
**Ligand Expo**	http://ligand-expo.rcsb.org/	Ligand Expo is a resource offering chemical and structural information about small molecules found within the Protein Data Bank entries, along with tools for searching, identifying entries with specific molecules, downloading 3D molecule structures, and creating new chemical definitions.	[113]
***Databases offering diverse types of information***			
**LINCS**	https://lincsproject.org/	The LINCS Consortium is a project that provides public data on cellular responses to various genetic and environmental stressors, aiming to deepen our understanding of cellular pathways and aid in the development of therapies to normalize disturbed pathways and networks, with their website and data portal offering comprehensive information on assays, cell types, perturbations, and related software for data analysis.	[114]
**BRENDA**	http://www.brenda-enzymes.org/	BRENDA is a comprehensive resource that consolidates extensive information about enzymes and enzyme–ligand relationships derived from various sources and offers adaptable search systems and assessment tools.	[115]
**COCONUT**	https://coconut.naturalproducts.net	Natural Products Online is a freely accessible, open-source platform dedicated to storing, searching, and analysis of natural products (NPs). It currently features COCONUT, a comprehensive and well-documented collection of open natural products, which is one of the most significant resources available without any restrictions.	[116]
**TDR targets**	https://tdrtargets.org	TDR Targets is a website that serves two purposes. Firstly, it provides information on targets, drugs, and bioactive compounds. Secondly, it can be used to prioritize targets within whole genomes.	[117]

* All URL addresses were accessed on 3 September 2023.

**Table 3:** AI-based methodologies to predict DTIs.

Approach	Year	Datasets	Features	Algorithms	Performance	Ref.
DTiGEMS+	2020	The literature [173]	Similarity-based features	Graph embedding, graph mining, similarity network fusion, MLP, RF, Adaboost	AUPR of 0.88, 0.86, 0.96, and 0.97 for the NR, GPCR, IC, and E datasets	[174]
GanDTI	2021	DUD-E [175], bindingDB inhibition, the literature [176,177]	Molecule fingerprints with a radius of two, protein data encoded overlapping amino acid sequences	GNN, attention mechanism to formulate summarized protein feature vectors, MLP	AUC of 0.983, Recall of 0.933 and Precision of 0.960	[178]
DTI prediction using multiple kernel-based triple collaborative matrix factorization	2022	DrugBank, BRENDA, SuperTarget [179], KEGG BRITE	Gaussian interaction profile, network of drug-side effect associations, MACCs drug substructure fingerprint, and chemical structure for drug kernels, Gaussian interaction profile for target, PPIs network of target, functional information of target and sequence information of target for target kernels	Multiple kernel-based triple collaborative matrix factorization (MKTC-MF)	AUPR of 0.933 on ion channel	[180]
DeepFusion	2022	BIOSNAP [181], DAVIS dataset [182]	Global structural similarity feature based on similarity theory and convolutional neural network for both drug and protein, local chemical sub-structure semantic feature using transformer network for both drug and protein	Deep-learning-based multi-scale feature fusion method including CNN and transformer network	Best ROC-AUC of 0.911	[183]
AttentionSiteDTI	2022	Protein Data Bank, DUD-E, human dataset from Liu et al. [176], BindingDB	Graph-based features of proteins and drugs	Topology adaptive graph CNN (TAGCN), MLP, self-attention mechanism, bidirectional long short-term memory (LSTM)	Best AUC of 0.991 in human dataset	[184]
MINN-DTI	2022	DUD-E, human dataset from Liu et al. [176], BindingDB	A 2D distance map for the target and the 2D molecular graph for the molecule	Dynamic CNN (DyCNN), inter-CMPNN, MLP	Best AUC of 0.967 in human dataset	[185]
MDTips	2023	Drug repurposing knowledge graph (DRKG), DrugBank, UniProt	Knowledge graph, drug-structure-based feature, target amino-acid-sequence-based feature, drug perturbation signatures, gene over-expression signatures, gene knockout/knockdown signature	Attentive FP and transformer encoders, knowledge graph embedding, ConvE, GAT, GNN, CNN, GCN	AUPR: 0.951 ± 0.003	[186]

**Table 4:** AI-based methodologies to predict various toxicity.

Approach	Year	Datasets	Features	Algorithms	Performance	Ref.
ToxicBlend	2019	Tox21 data, ToxCast	Physical chemicals descriptors, PubChem molecular fingerprints, SMILES n-grams	Multi-task XGBoost, multi-task NNs, graph convolutional model	AUC of 0.866 in Tox21 by random splits, AUC of 0.763 in ToxCast by scaffold splits	[222]
CEM-DNN	2023	ClinTox [223], Tox21, RTECS [224]	Morgan fingerprints, SMILES embeddings (SE)	Single-task DNN, multi-task DNN	AUC-ROC: 0.991 ± 0.011, balanced accuracy: 0.963 ± 0.028	[225]
admetSAR2.0	2019	DrugBank, ChEMBL, CPDB [226], Tox21, CYP450 dataset [227]	RDKit, Morgan, atom pairs, torsions, MACCS, SubFP fingerprints	kNN	AUC ranging from 0.625 to 0.992, with an average of 0.842	[228]
Interpretable-ADMET	2022	ChEMBL, PubChem, DrugBank, publications in the literature	Matched molecular pair (MMP)-processed fingerprint	Graph convolutional neural network (GCNN), graph attention network (GAT)	AUC of 0.977 in GCNN, AUC of 0.974 in GAT	[229]
HelixADMET	2022	ZINC15, DrugBank, ChEMBL, CPDB, Tox21, CYP450, PubChem assays	Subgraph (local structure) of a compound, molecular 3D conformation, molecular fingerprints	GNN, RF	AUC range of 0.803 to 0.967	[230]
Prediction and mechanistic analysis of DILI based on chemical structure	2021	DILIrank [231], SIDER	ECFP4 fingerprints, predicted protein targets, Mordred molecular descriptors	SVM, RF	Mean balanced accuracy of 0.759 ± 0.027	[232]
DILI prediction by maximizing fidelity through explicit subgraph feature mining	2022	DILIst [233], TDC [234]	SMILES converted to RDKit mol and networkx graph object	Supervised subgraph mining (SSM)	AUC: 0.691, F1-score: 0.784, MCC: 0.338	[235]
deephERG	2019	ChEMBL	Mol2vec, 2D MOE descriptors	Multitask DNN	Best AUC: 0.967, 29.6% of FDA-approved drugs potentially possessed hERG inhibitory activity	[31]
Cardiotoxicity prediction of Artemisinin derivatives	2021	PubMed, PubChem, DrugBank	The calculated descriptors	RF	AUC greater than 0.830 for cardio-toxicity parameters	[236]
Predicting mutagenicity in pyrrolizidine alkaloids	2021	The literatures [237,238,239], EFSA dataset [240]	MolPrint2D fingerprints, chemistry development kit	Lazar with high confidence, all lazar predictions, RF, logistic regression (stochastic gradient descent), logistic regression (scikit), NN, SVM	Accuracies of 80–85%	[241]
Carcinogenic classification using a triple classification prediction model	2023	Inventory of Hazardous Chemicals [242], Globally Harmonized System of Classification and Labeling of Chemicals (GHS) [243]	Generated by calculation and RF feature selection, including AATSC0p and GATS1e	MLP. XGBoost. kNN, complement naïve Bayes, SVM, LR. RF	The best accuracy in IARC dataset by RF	[244]

## Data Availability

Data sharing is not applicable.
